# Quality of life profile in three cohorts of community-dwelling Swiss older people

**DOI:** 10.1186/s12877-019-1112-4

**Published:** 2019-04-02

**Authors:** Nazanin Abolhassani, Brigitte Santos-Eggimann, Christophe Büla, René Goy, Idris Guessous, Yves Henchoz

**Affiliations:** 1 Center for Primary Care and Public Health (Unisanté), University of Lausanne, Biopôle 2, SV-A, Bio2–00–161, Route de la Corniche 10, 1010 Lausanne, Switzerland; 2 Service of Geriatric Medicine and Geriatric Rehabilitation, Lausanne university hospital, Lausanne, Switzerland; 3 Pro Senectute Vaud, Lausanne, Switzerland; 4 Unit of Population Epidemiology, Department of Community Medicine and Primary Care and Emergency Medicine, University Hospital of Geneva, Geneva, Switzerland

**Keywords:** Quality of life, Satisfaction, Importance, Community older people, Cohort

## Abstract

**Background:**

Quality of life (QoL) is a subjective and dynamic concept resulting from an interplay between importance of and satisfaction with different aspects of life. However, it is unclear whether social contexts experienced by individuals born at specific times in history (cohort effects) may influence QoL in old age. This study aimed to compare among older persons born before, during, and at the end of World War II: a) satisfaction with QoL, overall and per domains; b) importance of QoL domains.

**Methods:**

This repeated cross-sectional study included representative samples of community-dwelling adults born in 1934–1938 (pre-war), 1939–1943 (war), and 1944–1948 (baby-boom) from the Lausanne cohort 65+. QoL was assessed overall, and in seven domains in 2011 and 2016. Two-by-two cohort comparisons were performed at ages 68–72 (war versus baby-boom) and 73–77 years (pre-war versus war).

**Results:**

Overall satisfaction with QoL did not differ between cohorts despite increased education level across cohorts and a shift between pre-war and war cohorts towards lower morbidity and higher proportion living alone. However, “Feeling of safety” consistently showed significant improvements from earlier to later-born cohorts. Furthermore, the war cohort reported higher satisfaction than pre-war cohort in “Autonomy”. Conversely, no significant difference was observed between cohorts in importance of QoL domains, except increased importance given to “Health and mobility” in the war compared to pre-war cohort.

**Conclusions:**

Societal changes reflected in the profile of successive elders’ cohorts did not appear to modify the overall satisfaction with QoL.

**Electronic supplementary material:**

The online version of this article (10.1186/s12877-019-1112-4) contains supplementary material, which is available to authorized users.

## Background

With population aging and increased life expectancies in high-income countries, quality of life (QoL) has become important. Since influences of QoL have expanded beyond the personal concerns for health to the society and to policy interest in the potential for reducing public expenditure, several social and health aspects should be considered while addressing QoL in older people [1–4]. Furthermore, unlike the previous dominant paradigm of QoL decline in old age [5] due to higher risk of cognitive and physical impairments as well as of social losses [6, 7], some empirical research indicated no age-related decline in QoL and greater satisfaction with life than in younger age groups [7–9]. These observations may reflect a shift of emphasis away from the previously negative construction of old age [10] and then the question, ‘if contextual factors influence QoL in different older generations at different ages?’ has gained relevance that warrants further investigation into the complex concept of QoL.

QoL is generally viewed as a multidimensional concept reflecting macro-societal and micro-individual influences and arising from the subjective feeling of satisfaction or dissatisfaction in many domains important to people [4, 11–14]. The level of satisfaction with these domains is therefore a function of its perceived importance by the individual [15]. As a consequence, QoL appears more as a dynamic concept with significant within-subject and between-subjects variations [16]. Within-subject differences refer to changes in an individual’s QoL over his/her life span [17]. Between-subjects differences result from variations in QoL among different groups defined by characteristics such as age [18], gender [12] or belonging to specific birth cohorts and thus ageing in specific socio-economic contexts [17].

To get further insight on the dynamic interplay between importance, satisfaction and QoL, we investigated the hypothesis that different social contexts experienced by individuals born at specific times in history may determine cohort effects (also referred to as “generation” effects) [19] on QoL through variations in satisfaction with, as well as in perceived importance of QoL domains. The specific socio-economic contexts caused by World War II, as a macro-societal influence, even in countries not directly involved in the conflict [20], offered a unique opportunity to assess variations in older people’s QoL across successive birth cohorts. Thus, this study aimed to measure and compare among community-dwelling older persons born before, during, and after World War II: a) their satisfaction with QoL, overall and per domains; b) the importance they gave to each domain of QoL.

## Methods

### Study population and design

Data was drawn from the Lausanne cohort 65+ (Lc65+), a longitudinal, observational study investigating age-related frailty among persons aged 65 years and over living in Lausanne, Switzerland. Detailed descriptions of the study design have been reported elsewhere [21]. In brief, three representative samples of the community-dwelling population of Lausanne city enrolled at the age of 65 to 70 were randomly selected in 2004 (pre-war cohort), 2009 (war cohort), and 2014 (baby-boom cohort). Eligibility was defined by the place of residence (Lausanne, a Swiss city of 125,000 inhabitants) and by the year of birth. Subjects living in an institution or unable to respond by themselves due to advanced dementia were excluded. Compared to the total population of Lausanne, participants to the Lc65+ study in 2016 did not differ in gender or in birth year distributions (Additional file 1: Table S1)**.** Furthermore, the socio-economic characteristics of participants enrolled in 2004 closely reflected the Lausanne general population in the same age category in aggregate statistics from the Population Office or from the 2000 Swiss national population census [21].

The current study focused on surviving, non-institutionalized participants still living in Lausanne who completed a QoL assessment in 2011 and 2016 in-person (proxy-reports excluded). From 1′564 pre-war subjects enrolled in 2004, 1′108 (70.8%) were eligible for QoL assessment in 2011 and 1′078 (97.3% of eligible) responded. From 1′489 war subjects enrolled in 2009, 1′351 (90.7%) were eligible for QoL assessment in 2011 and 1′264 (93.6% of eligible) responded; 1′077(72.3%) were still eligible for QoL assessment in 2016 and 1′041 (96.7% of eligible) responded. Finally, from 1′678 baby-boomers enrolled in 2014, 1′493 (89.0%) were eligible for QoL assessment in 2016 of whom 1′381 (92.5% of eligible) responded (Additional file 2**:** Figure S1).

The study protocol was approved by the Ethics Committee of the Faculty of Biology and Medicine of the University of Lausanne (Protocol No. 19/04).

### Data collection

Required data for the current study (sociodemographic, health and quality of life related data) were collected through postal questionnaires.

#### Sociodemographic and health related measures

Socio-demographic data included gender; educational level (at time of enrolment) categorized according to the International Standard Classification of Education (ISCED) [22] as low (obligatory school or ISCED 0–2), medium (apprenticeship or ISCED 3), or high (college, university degree or equivalent or ISCED 4–8); and living arrangement (alone vs. not alone). Health related data included medical diagnoses and depressive symptoms. For medical diagnoses, the participants were asked whether they suffered from or received treatment for any of the twelve following selected health conditions or diseases, diagnosed by a physician, over the last 12-month period: hypertension, myocardial ischemia, other heart disease, stroke, diabetes, chronic lung disease, asthma, osteoporosis, arthrosis or arthritis, malignant neoplasm, ulcer and Parkinson’s disease. The number of reported medical diagnoses was categorized into three groups (“zero”, “one”, “two or more”). For depressive symptoms, the participants were asked the following questions of the Primary Care Evaluation of Mental Disorders Procedure: “During the past month, have you often been bothered by 1) feeling down, depressed, or hopeless? ; 2) Little interest in doing things?” A positive answer to any or both of the two questions was defined as the presence of depressive symptoms [23].

#### Importance of QoL domains

QoL was assessed in 2011 and 2016 using a written, self-administered 28-item questionnaire covering health, social, cultural and economic factors (Additional file 3: Table S2)**.** This 28-item questionnaire was developed by a research group on the quality of life of older people [24], based on the available evidence, including the World Health Organization report on social determinants of health [25], the synthesis of the literature [26] and the experience of the group’s members. A factorial structure, consisting of seven QoL domains, was previously explored and validated with sufficient internal consistency within each domain (“Material resources”, “Close entourage”, “Social and cultural life”, “Esteem and recognition”, “Health and mobility”, “Feeling of safety” and “Autonomy”). This factorial structure was highly consistent between an exploratory and a validation sample, with adequate internal consistency within each domain [24].

Participants were first asked to rate the importance of each item on their own QoL (0 = very low; 1 = quite low; 2 = quite high; 3 = very high). The importance score of each domain was computed through summing up the ratings of constituent items, dividing by the maximum possible score (number of constituent items multiplied by three), and then multiplying by 100 to obtain a score (range: 0–100). The importance scores for QoL domains with more than one missing constituent item within each domain were treated as missing.

#### Satisfaction with QoL domains

To assess satisfaction with the QoL domains, participants were asked to rate their perceived discomfort or dissatisfaction (0 = not at all, 1 = a little, 2 = a lot) for each of the 28 items. Using reverse coding, an overall score of satisfaction with QoL was calculated by summing answers to all 28 QoL items, dividing by 56 (maximum possible score), and multiplying by 100. The overall score of satisfaction with QoL with more than half of the constituent items missing (i.e. more than 14 of 28) was treated as missing.

Similarly, a satisfaction score was calculated for each domain through summing up the ratings of constituent items, dividing by the maximum possible score (number of constituent items multiplied by two), and then multiplying by 100 to obtain a score ranging from 0 to 100. The satisfaction scores for QoL domains with more than one missing constituent item within each domain were treated as missing.

#### Statistical analysis

**Fig. 1:**
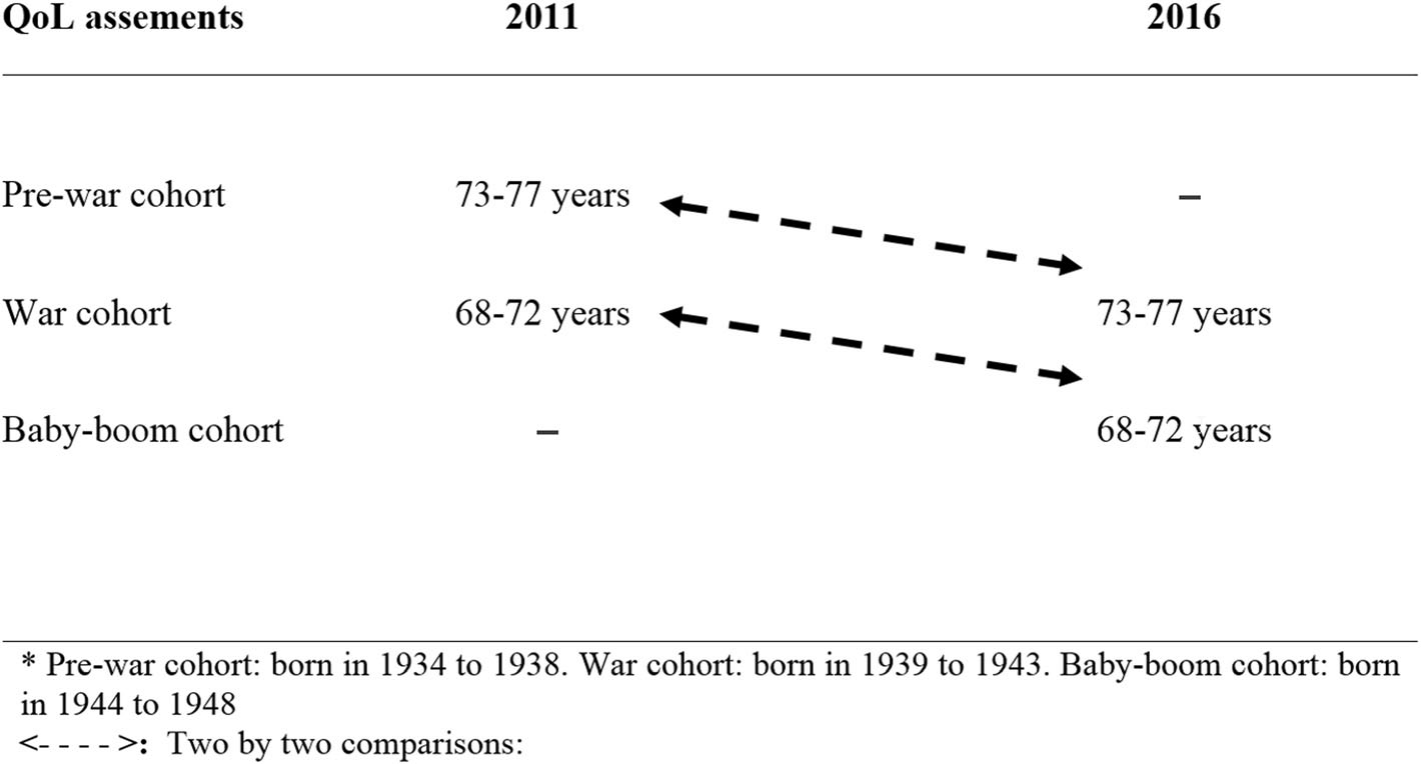
Age at quality of life assessments in pre-war, war and baby-boom cohorts^*^

Descriptive statistics were used to present sample characteristics. Results were expressed as number of participants (percentage) for categorical variables (sociodemographic and health related data) and as mean ± standard deviation for QoL data. As illustrated in Fig. 1, we compared two-by-two the cohorts’ profile at age 68–72 years (pre-war cohort’s 2011 assessment versus baby-boom cohort’s 2016 assessment) and at age 72–77 years (pre-war cohort 2011 versus war cohort 2016). Bivariate analysis was performed using chi square test for categorical variables; mean scores of importance of QoL domains, of satisfaction with QoL domains, and overall score of satisfaction with QoL were compared using the t-test. To check any cohort effect, considering the overall score of satisfaction with QoL as outcome, linear regression analyses were performed for the age groups separately adjusting for control variables including gender, educational level category, living arrangement, medical diagnoses and depressive symptoms. Statistical significance was considered for a two-side test with *p* < 0.05. All statistical analyses were performed using Stata software version 15.0 (Stata Corp, College Station, TX, USA).

## Results

### Characteristics of participants

Descriptive characteristics of included participants are summarized in Table 1. In all three cohorts and at both time points, the majority were female, with middle or high education and cohabiting. Education level differed significantly across cohorts both at age 68–72 years (*P* = 0.002) and at age 73–77 years (*P* < 0.001), with higher education in later-born cohorts. A difference in living arrangement was apparent only at age 73–77 years, with a higher proportion of the war than the pre-war cohort reporting to live alone (*P* = 0.047). At age 68–72 years, about a quarter of participants in the war and baby-boom cohorts reported two or more medical diagnoses. At age 73–77 years, more than a third reported two or more medical diagnoses in the pre-war and war cohorts and there were significant differences in the number of medical diagnoses between the pre-war and war cohorts (*P* = 0.004) with a higher level of medical diagnoses in the pre-war cohort. Finally, the proportion of participants reporting depressive symptoms did not differ across cohorts, but was slightly higher at age 73 to 77 years than at age 68 to 72 years.

**Table 1:** Characteristics of participants at age 68–72 years and 73–77 years, by period of assessment (cohort)*

	Age 68–72 years			Age 73–77 years		
	2011 (war)	2016 (baby-boom)	*P*-value	2011 (pre-war)	2016 (war)	*P*-value
	*N* = 1264	*N* = 1381		*N* = 1078	*N* = 1041	
Gender			0.178			0.762
Men	521 (41.2)	605 (43.8)		418 (38.8)	397 (38.1)	
Women	743 (58.8)	776 (56.2)		660 (61.2)	644 (61.9)	
Education			0.002			< 0.001
High	533 (42.2)	663 (48.1)		382 (35.6)	446 (42.8)	
Medium	506 (40.0)	525 (38.0)		432 (40.3)	414 (39.8)	
Low	225 (17.8)	192 (13.9)		258 (24.1)	181 (17.4)	
Living arrangement			0.361			0.047
Not alone	755 (59.9)	846 (61.7)		633 (59.0)	569 (54.7)	
Alone	505 (40.1)	526 (38.3)		440 (41.0)	471 (45.3)	
Medical diagnoses			0.613			0.004
0	472 (37.6)	543 (39.5)		275 (25.5)	298 (28.6)	
1	454 (36.2)	483 (35.2)		368 (34.1)	395 (37.9)	
2 or more	328 (26.2)	348 (25.3)		435 (40.4)	348 (33.5)	
Depressive symptoms			0.349			0.483
No	956 (76.4)	1068 (78.0)		782 (72.8)	772 (74.2)	
Yes	295 (23.6)	302 (22.0)		292 (27.2)	269 (25.8)	

Results are expressed as number (%).

*Pre-war cohort: born in 1934 to 1938. War cohort: born in 1939 to 1943. Baby-boom cohort: born in 1944 to 1948

#### Overall score of satisfaction with QoL

Overall scores of satisfaction with QoL (mean ± standard deviation), in the whole sample and per sociodemographic and health subgroups, are presented in Table 2. Overall satisfaction with QoL did not differ either between the war and baby-boom cohorts at age 68–72 years or between the pre-war and war cohorts at age 73–77 years, including after stratification by gender, education, number of medical diagnoses and depressive symptoms. Only among the subgroups cohabiting at 73–77 years, satisfaction with QoL was significantly lower (*P* = 0.002) in the pre-war cohort compared to the war cohort.

**Table 2:** Score of overall satisfaction with QoL (mean ± SD) at age 68–72 years and 73–77 years, in whole sample and per subgroups, by period of assessment (cohort)*

	Age group 68–72 years			Age group 73–77 years		
	2011 (war)	2016 (baby-boom)	P-value	2011 (pre-war)	2016 (war)	*P*-value
	*N* = 1264	*N* = 1381		*N* = 1078	*N* = 1041	
Whole sample	88.1 ± 15.4	88.1 ± 16.4	0.926	86.1 ± 16.9	87.1 ± 16.3	0.174
Gender						
Men	88.0 ± 16.8	88.4 ± 15.7	0.645	87.2 ± 16.0	87.8 ± 17.1	0.634
Women	88.2 ± 14.3	87.9 ± 16.8	0.767	85.4 ± 17.5	86.7 ± 15.8	0.165
Education						
High	90.3 ± 12.2	90.6 ± 12.2	0.672	89.3 ± 12.8	89.4 ± 13.8	0.961
Medium	87.9 ± 15.5	87.6 ± 17.7	0.785	85.6 ± 17.7	86.4 ± 17.0	0.470
Low	83.0 ± 20.2	80.8 ± 22.2	0.316	82.5 ± 19.8	83.0 ± 19.2	0.794
Living arrangement						
Not alone	89.4 ± 14.8	89.9 ± 15.0	0.463	86.6 ± 17.0	89.4 ± 14.5	0.002
Alone	86.2 ± 16.0	85.4 ± 17.9	0.449	85.5 ± 16.7	84.4 ± 17.8	0.353
Medical diagnoses						
0	89.5 ± 15.0	90.7 ± 14.7	0.185	88.7 ± 16.2	90.0 ± 14.1	0.310
1	88.4 ± 15.1	88.1 ± 17.1	0.815	86.2 ± 17.7	87.6 ± 16.4	0.249
v2 or more	85.6 ± 16.2	84.1 ± 17.2	0.249	84.4 ± 16.5	84.0 ± 17.4	0.746
Depressive symptoms						
No	90.7 ± 13.2	90.9 ± 14.3	0.788	88.8 ± 16.1	90.2 ± 13.8	0.070
vYes	79.4 ± 18.6	78.3 ± 19.5	0.466	78.6 ± 16.9	78.2 ± 19.3	0.805

*Pre-war cohort: born in 1934 to 1938. War cohort: born in 1939 to 1943. Baby-boom cohort: born in 1944 to 1948

Furthermore, in a multivariate analysis predicting the overall score of satisfaction with QoL that adjusted for gender, educational level, living arrangement, number of medical diagnoses and depressive symptoms, there was still no significant cohort effect in any of the two age groups.

#### Scores of satisfaction and importance in QoL domains

Scores of satisfaction in each of the seven QoL domain (mean ± standard deviation) are presented in Table 3. “Feeling of safety” was the only domain that, at both age groups (*P* = 0.035 at age 68–72 years and *P* = 0.038 at age 73–77 years), consistently showed significant improvements in satisfaction from earlier to later-born cohorts. An improvement was also observed (*P* ≃ 0.050) in the “Autonomy” domain in the more recently born cohort, but only at the age of 73–77 years.

**Table 3:** Scores of satisfaction with QoL domains at age 68–72 years and 73–77 years, by period of assessment (cohort)*

Domains	Age group 68–72 years			Age group 73–77 years		
	2011 (war)	2016 (baby-boom)	P-value^**^	2011 (pre-war)	2016 (war)	*P*-value^**^
	*N* = 1264	*N* = 1381		*N* = 1078	*N* = 1041	
Material resources	87.7 ± 18.7	87.7 ± 18.4	0.996	87.2 ± 19.4	87.7 ± 19.3	0.555
Close entourage	86.8 ± 19.8	86.9 ± 20.2	0.972	86.3 ± 20.7	86.4 ± 19.8	0.913
Social & cultural life	86.7 ± 18.4	86.7 ± 19.7	0.960	84.3 ± 20.2	85.2 ± 19.7	0.350
Esteem & recognition	84.9 ± 23.8	84.4 ± 25.1	0.570	82.8 ± 25.9	84.0 ± 24.6	0.313
Health & mobility	89.8 ± 19.5	89.3 ± 20.7	0.501	86.5 ± 21.8	87.1 ± 22.0	0.560
Feeling of safety	86.5 ± 20.5	88.2 ± 20.6	0.035	84.6 ± 21.5	86.6 ± 20.8	0.038
Autonomy	91.5 ± 18.4	91.5 ± 19.5	0.945	89.8 ± 20.3	91.5 ± 18.2	0.050

* Pre-war cohort: born in 1934 to 1938. War cohort: born in 1939 to 1943. Baby-boom cohort: born in 1944 to 1948

** Results are expressed as mean ± standard deviation and between surveys comparisons using t-test

Scores of importance for each domain of QoL (mean ± standard deviation) are presented in Table 4. We observed no significant difference between cohorts in the mean score of importance attributed to any domain at age 68–72 years or 73–77 years, except for the “Health and mobility” domain where at age 73 to 77 years the later-born cohort reported a higher level of importance.

**Table 4:** Scores of the importance of QoL domains at age 68–72 years and 73–77 years, by period of assessment (cohort)*

Domains	Age group 68–72 years			Age group 73–77 years		
	2011 (war)	2016 (baby-boom)	P-value^**^	2011 (pre-war)	2016 (war)	*P*-value^**^
	*N* = 1264	*N* = 1381		*N* = 1078	*N* = 1041	
Material resources	68.9 ± 15.4	69.4 ± 14.9	0.392	69.1 ± 15.3	69.9 ± 16.1	0.201
Close entourage	72.1 ± 19.1	72.4 ± 18.2	0.678	72.4 ± 18.8	71.9 ± 18.7	0.569
Social & cultural life	57.6 ± 20.3	58.4 ± 19.7	0.282	57.2 ± 21.3	58.2 ± 20.3	0.266
Esteem & recognition	70.0 ± 19.6	71.1 ± 18.8	0.134	69.0 ± 19.3	69.9 ± 20.0	0.319
Health & mobility	85.2 ± 17.0	84.9 ± 16.4	0.655	82.1 ± 18.1	83.7 ± 17.5	0.034
Feeling of safety	80.4 ± 17.6	79.1 ± 17.5	0.065	80.2 ± 17.8	80.9 ± 17.6	0.312
Autonomy	80.4 ± 15.9	80.2 ± 14.7	0.834	78.8 ± 16.2	79.7 ± 16.5	0.238

* Pre-war cohort: born in 1934 to 1938. War cohort: born in 1939 to 1943. Baby-boom cohort: born in 1944 to 1948

**Results are expressed as mean ± standard deviation and between surveys comparisons using t-test

## Discussion

This population-based study addressed cohort effects on the overall satisfaction with QoL as well as on satisfaction with, and importance of, seven QoL domains in community-dwelling older people, taking the World War II as a pivotal historical event determining socioeconomic life circumstances. Despite significant differences in cohorts’ profile (improvements in education in later-born cohorts, lower morbidity profile in the war cohort who more frequently lived alone than the previous cohort), no significant change on their respective satisfaction with QoL was observed at the ages of 68–72 years and 73–77 years. This holds true when further analysing subgroups of the population, with only one exception among older persons still living with others at the age of 73–77 years who appeared more satisfied when born during rather than before WWII. An important contribution of this study is thus to question the assumption that stability of the overall QoL across birth cohorts might result from internal compensation of positive and negative changes. Indeed, results from the present study also reveal a significant improvement in satisfaction with QoL only for the “Feeling of safety” domain in the later-born cohorts. No other cohort effect was observed in satisfaction with QoL for any other specific QoL domains.

This study also provides unique information on changes in the importance attributed to QoL domains across cohorts and at different ages. Our initial hypothesis that cohort differences in educational, socio-economic, and health profiles might translate into changes in the importance attributed to QoL domains was not confirmed. Furthermore, we found only very limited evidence that these changes in profiles could modify and compensate possible differences in satisfaction with QoL. Indeed, only the “Health and mobility” domain appeared to have a significantly higher importance in QoL at age 73–77 year among participants from the war cohort who, on the other hand, reported a lower level of medical diagnoses. Overall, these results emphasize the complexity of the relationship between changing life circumstances and QoL. Further longitudinal analyses among the same cohort over time are needed to further disentangle the issue of between and within subject’s change in QoL satisfaction over time. The within subject dynamism of the QoL concept involves a ‘response shift’ phenomenon resulting from an evolution in the respondent’s values (reprioritization of the importance attributed to the component domains) [27–29]. What was once important may seem unimportant and what once ignored may have great importance [30]. Such response shift may also occur at the population-level [31].

Results of the current study also challenge those from previous studies. While the observed different socio-economic and health profiles of successive cohorts did not influence their satisfaction with QoL in our analysis, a study on life satisfaction trajectories of older women living in Switzerland reported that the mean life satisfaction score and the prevalence of satisfied women were lower in more recent cohorts of identical ages [17]. However, in this study, only women were investigated and cohorts were defined with a 10-year interval, irrespective of historical events.

Results from the current study might also be related to previous observations suggesting that satisfaction with QoL is influenced by expectations [32], resulting in a stability-despite-loss paradox or ‘satisfaction paradox’ [7, 33]. In other words, on one hand, as the more recent cohorts benefited from better socio-economic contexts, they are expected to better cope with aging and to express higher satisfaction with life. On the other hand, difficulties experienced by earlier cohorts may translate into lowered expectations, resulting in more satisfaction than the next generations would feel in similar circumstances [34]. Such adaptation can be also described in terms of response shift, by which individuals change their internal standards, values and conceptualizations of QoL to accommodate some hardship or negative circumstance [27]. Closely allied to adaptation is fostered resilience, the phenomenon of people beating the odds and doing well against expectation [3, 35] that may be a reason for such favorable perception of QoL; and a study by Seery et al. indicated that people with some prior lifetime adversity were the least affected by recent adverse events [36]. Finally, satisfaction with QoL is influenced by social comparisons [32], mainly downward contrast comparisons and the advantage of “counting blessings” and “not complaining” as coping strategies may also play a role in preserving perceived QoL in individuals experiencing a declining situation [37–39].

### Strengths and limitations

This study took advantage of an historical event that determined specific socio-economic contexts in which each of the successive cohorts were born (before, during, and end of World War II), grew up and worked. This afforded a unique opportunity to assess variations in both the importance attributed to the multiple facets of QoL and the level of satisfaction with them between the cohorts at a given age.

A limitation of this study could potentially be the difficulty to disentangle cohort and period effects. However, no major event occurred in Switzerland between 2011 and 2016 that would influence responses to the QoL assessments. We also relied on a relatively short time interval (5 years) to define the birth cohorts, which may have limited the ability to observe differences; however, results pointed to significant socio-economic and health differences between cohorts that could have likely had an impact on their QoL in spite of this short interval.

## Conclusion

Despite certain differences in socio-economic, educational, and health profiles between cohorts in identical age groups, no difference was observed in the overall satisfaction with QoL as well as in the scores of importance and satisfaction in most QoL domains.

## Additional files

Additional file 1: **Table S1.** Comparison of gender and birth year distributions between the population of Lausanne and participants to the Lc65+ study in 2016. To provide information on the representativeness of the Lc65+ cohort, gender and birth year distributions between the population of Lausanne (permanent resident population of Lausanne on 31st December 2016) and participants to the Lc65+ study in 2016 were compared separately per pre-war, war and baby boom cohorts. (PDF 96 kb)

Additional file 2: **Figure S1.** selection procedure of participants. Three representative samples of the community-dwelling population of Lausanne city enrolled at the age of 65 to 70 were randomly selected in 2004 (pre-war cohort/ born 1934–1938), 2009 (war cohort/ born 1939–1943), and 2014 (baby-boom cohort/ born 1944–1948). The current study focused on surviving, non-institutionalized participants still living in Lausanne who completed a QoL assessment in 2011 and 2016 in-person (proxy-reports excluded). From 1′564 pre-war subjects enrolled in 2004, 1′108 (70.8%) were eligible for QoL assessment in 2011 and 1′078 (97.3% of eligible) responded. From 1′489 war subjects enrolled in 2009, 1′351 (90.7%) were eligible for QoL assessment in 2011 and 1′264 (93.6% of eligible) responded; 1′077(72.3%) were still eligible for QoL assessment in 2016 and 1′041 (96.7% of eligible) responded. Finally, from 1′678 baby-boomers enrolled in 2014, 1′493 (89.0%) were eligible for QoL assessment in 2016 of whom 1′381 (92.5% of eligible) responded (DOCX 14 kb)

Additional file 3: **Table S2.** List of 28 quality of life items. This 28-item questionnaire was developed by the Research Group on the quality of life of older people in cantons of Vaud and Geneva (Switzerland), based on the available evidence, including the World Health Organization report on social determinants of health and the synthesis of the literature as well as the experience of the Research Group in this field. The questionnaire reflects the convergence of health, social, cultural and economic factors of older people’s QoL. (DOCX 20 kb)

## Abbreviations

ISCED: International Standard Classification of Education
Lc65 +: Lausanne cohort 65+
QoL: Quality of life

## References

[1] Hjorthol RJ, Levin L, Sirén A (2010). Mobility in different generations of older persons: the development of daily travel in different cohorts in Denmark, Norway and Sweden. J Transp Geogr.

[2] Dale CE, Bowling A, Adamson J, Kuper H, Amuzu A, Ebrahim S (2013). Predictors of patterns of change in health-related quality of life in older women over 7 years: evidence from a prospective cohort study. Age Ageing.

[3] Netuveli G, Blane D (2008). Quality of life in older ages. Br Med Bull.

[4] Bowling A, Banister D, Sutton S, Evans O, Windsor J (2002). A multidimensional model of the quality of life in older age. Aging Ment Health.

[5] Higgs P, Hyde M, Wiggins R, Blane D (2003). Researching quality of life in early old age: the importance of the sociological dimension. Social Policy & Administration.

[6] Grundy E (2006). Ageing and vulnerable elderly people: European perspectives. Ageing & Society.

[7] Kunzmann U, Little TD, Smith J (2000). Is age-related stability of subjective well-being a paradox? Cross-sectional and longitudinal evidence from the Berlin aging study. Psychol Aging.

[8] Gwozdz W, Sousa-Poza A (2010). Ageing, health and life satisfaction of the oldest old: an analysis for Germany. Soc Indic Res.

[9] Dello Buono M, Urciuoli O, De Leo D (1998). Quality of life and longevity: a study of centenarians. Age Ageing.

[10] Von Dem Knesebeck O, Wahrendorf M, Hyde M, Siegrist J (2007). Socio-economic position and quality of life among older people in 10 European countries: results of the SHARE study. Ageing & Society.

[11] Brown J, Bowling A, Flynn T. Models of quality of life. A taxonomy and systematic review of the literature: European Forum on Population Ageing Research; 2004.

[12] Molzahn A, Skevington SM, Kalfoss M, Makaroff KS (2010). The importance of facets of quality of life to older adults: an international investigation. Qual Life Res.

[13] Sammarco A (2001). Perceived social support, uncertainty, and quality of life of younger breast cancer survivors. Cancer Nurs.

[14] Felce D, Perry J (1995). Quality of life: its definition and measurement. Res Dev Disabil.

[15] Hagell P, Westergren A (2006). The significance of importance: an evaluation of Ferrans and Powers' quality of life index. Qual Life Res.

[16] Allison PJ, Locker D, Feine JS (1997). Quality of life: a dynamic construct. Soc Sci Med.

[17] Burton-Jeangros C, Zimmermann-Sloutskis D (2016). Life satisfaction trajectories of elderly women living in Switzerland: an age–period–cohort analysis. Ageing & Society.

[18] Molzahn AE, Kalfoss M, Schick Makaroff K, Skevington SM (2011). Comparing the importance of different aspects of quality of life to older adults across diverse cultures. Age Ageing.

[19] Last JM, Abramson JH, Freidman GD (2001). A dictionary of epidemiology.

[20] Van Ewijk R, Lindeboom M (2017). Why people born during world war II are healthier.

[21] Santos-Eggimann B, Karmaniola A, Seematter-Bagnoud L, Spagnoli J, Bula C, Cornuz J (2008). The Lausanne cohort Lc65+: a population-based prospective study of the manifestations, determinants and outcomes of frailty. BMC Geriatr.

[22] UNESCO. (2011). http://www.uis.unesco.org/Education/Pages/international-standard-classification-of-education.aspx. Accessed 26 Oct 2016.

[23] Whooley MA, Avins AL, Miranda J, Browner WS (1997). Case-finding instruments for depression. Two questions are as good as many. J Gen Intern Med.

[24] Henchoz Y, Meylan L, Goy R, Guessous I, Bula C, Demont M (2015). Domains of importance to the quality of life of older people from two Swiss regions. Age Ageing.

[25] Wilkinson RG, Marmot M. Social determinants of health: the solid facts: World Health Organization; 2003.

[26] Kelley-Gillespie N (2009). An integrated conceptual model of quality of life for older adults based on a synthesis of the literature. Appl Res Qual Life.

[27] Sprangers MA, Schwartz CE (1999). Integrating response shift into health-related quality of life research: a theoretical model. Soc Sci Med.

[28] Schwartz CE, Sprangers MA (1999). Methodological approaches for assessing response shift in longitudinal health-related quality-of-life research. Soc Sci Med.

[29] Schwartz CE, Rapkin BD (2004). Reconsidering the psychometrics of quality of life assessment in light of response shift and appraisal. Health Qual Life Outcomes.

[30] Brown RI (1997). *Quality of life for people with disabilities: Models, research and practice*: Nelson Thornes.

[31] Lau D, Agborsangaya C, Al Sayah F, Wu X, Ohinmaa A, Johnson JA (2012). Population-level response shift: novel implications for research. Qual Life Res.

[32] Gabriel Z, Bowling A (2004). Quality of life from the perspectives of older people. Ageing & Society.

[33] Walker A (2005). A European perspective on quality of life in old age. Eur J Ageing.

[34] Brown J, Bowling A, Flynn T (2004). Models of quality of life: A taxonomy, overview and systematic review of the literature. European forum on population ageing research.

[35] Bartley M (2006). Capability and resilience: beating the odds. ESRC human capability and resilience research network.

[36] Seery MD, Holman EA, Silver RC (2010). Whatever does not kill us: cumulative lifetime adversity, vulnerability, and resilience. J Pers Soc Psychol.

[37] Gibbons FX (1999). Social comparison as a mediator of response shift. Soc Sci Med.

[38] Beaumont JG, Kenealy PM (2004). Quality of life perceptions and social comparisons in healthy old age. Ageing & Society.

[39] Wilhelmson K, Andersson C, Waern M, Allebeck P (2005). Elderly people's perspectives on quality of life. Ageing & Society.

