# Supplementary figures and images for: Quality of life profile in three cohorts of community-dwelling Swiss older people
Source: BMC Geriatr. 2019 Apr 2;19:96. doi: 10.1186/s12877-019-1112-4 (PMC6444620; doi:10.1186/s12877-019-1112-4)

**Supplementary figure 1: selection procedure of participants**

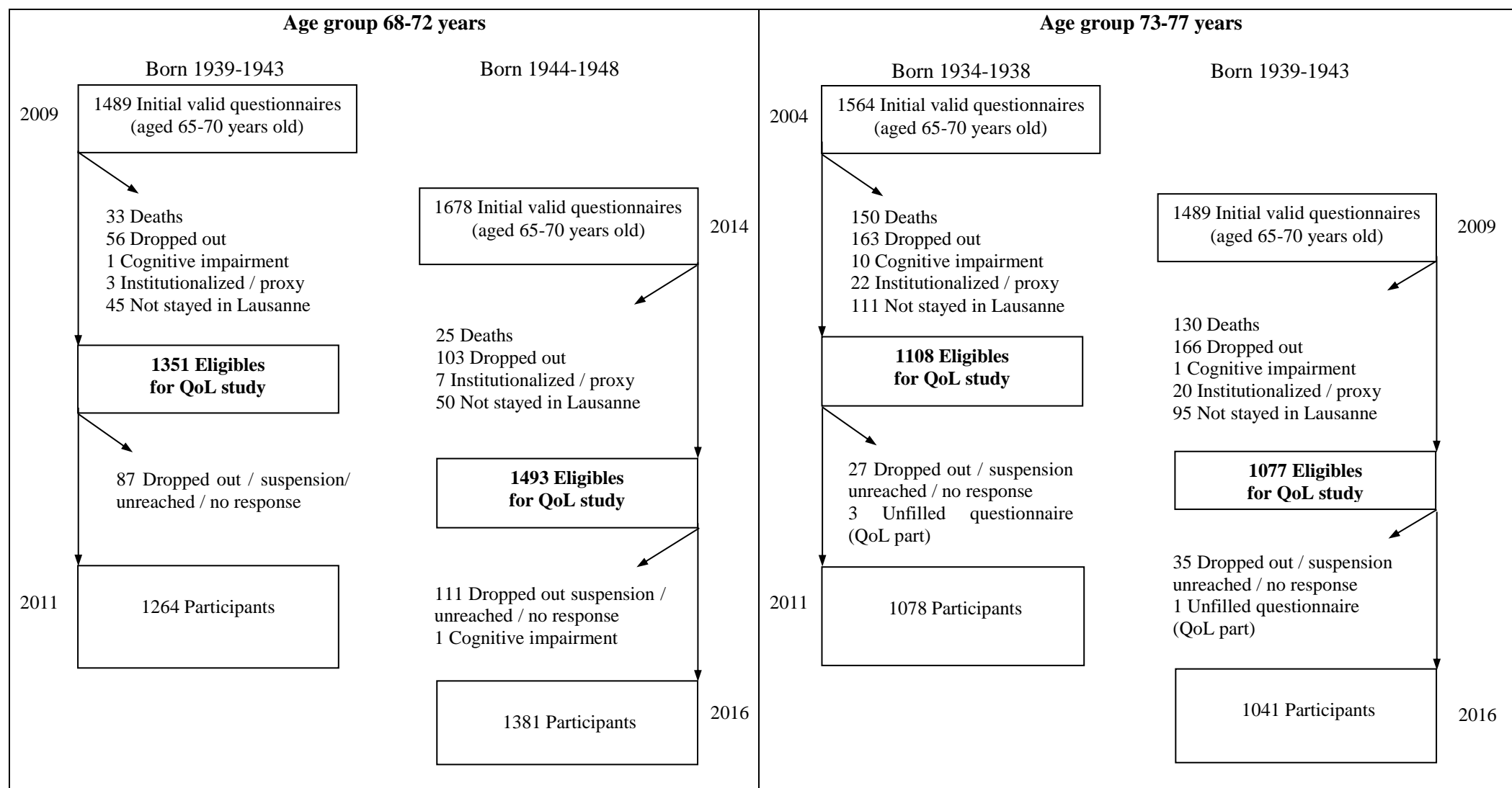

Supplement: Supplementary file 1 — Table S1. Comparison of gender and birth year distributions between the population of Lausanne and participants to the Lc65+ study in 2016. To provide information on the representativeness of the Lc65+ cohort, gender and birth year distributions between the population of Lausanne (permanent resident population of Lausanne on 31st December 2016) and participants to the Lc65+ study in 2016 were compared separately per pre-war, war and baby boom cohorts. (PDF 96 kb) [file 12877_2019_1112_MOESM1_ESM.pdf]
