# Supplementary material for: Quality of life profile in three cohorts of community-dwelling Swiss older people
Source: BMC Geriatr. 2019 Apr 2;19:96. doi: 10.1186/s12877-019-1112-4 (PMC6444620; doi:10.1186/s12877-019-1112-4)
Supplement: Supplementary file 2 — Figure S1. selection procedure of participants. Three representative samples of the community-dwelling population of Lausanne city enrolled at the age of 65 to 70 were randomly selected in 2004 (pre-war cohort/ born 1934–1938), 2009 (war cohort/ born 1939–1943), and 2014 (baby-boom cohort/ born 1944–1948). The current study focused on surviving, non-institutionalized participants still living in Lausanne who completed a QoL assessment in 2011 and 2016 in-person (proxy-reports excluded). From 1′564 pre-war subjects enrolled in 2004, 1′108 (70.8%) were eligible for QoL assessment in 2011 and 1′078 (97.3% of eligible) responded. From 1′489 war subjects enrolled in 2009, 1′351 (90.7%) were eligible for QoL assessment in 2011 and 1′264 (93.6% of eligible) responded; 1′077(72.3%) were still eligible for QoL assessment in 2016 and 1′041 (96.7% of eligible) responded. Finally, from 1′678 baby-boomers enrolled in 2014, 1′493 (89.0%) were eligible for QoL assessment in 2016 of whom 1′381 (92.5% of eligible) responded (DOCX 14 kb) [file 12877_2019_1112_MOESM2_ESM.docx]

**Supplementary table 2.** The 28-item quality of life questionnaire.

| Quality of Life domains | Item |
| --- | --- |
| *Feeling of safety* | Safety at home |
|  | Safety in the street |
|  | Adequate health insurance coverage |
|  | Access to health care and prevention |
| *Health and mobility* | Mobility, being able to travel alone |
|  | Being able to use public transport alone |
|  | Being able to travel |
|  | Not being dependent on help in daily life |
|  | Physical and mental health |
| *Autonomy* | Being able to express one's opinion, to vote, etc. |
|  | Being well informed to meet one's needs and decide |
|  | Being useful to others |
|  | Being able to manage money matters alone |
|  | Being able to decide on issues of daily life |
| *Close entourage* | Family relationships |
|  | Couples' relationships |
|  | Friendly atmosphere meals |
|  | Intergenerational relationships |
|  | Friendship relationships |
| *Material resources* | Housing comfort |
|  | Financial resources |
|  | Sufficient, good quality food |
| *Esteem and recognition* | Self-esteem |
|  | Being heard and respected |
| *Social and cultural life* | Integration into a group, association or society |
|  | Cultural and leisure activities |
|  | Religion, philosophy or spiritual life |
|  | Being able to exercise one's creativity, share ideas |
