# Supplementary material for: Quality of life profile in three cohorts of community-dwelling Swiss older people
Source: BMC Geriatr. 2019 Apr 2;19:96. doi: 10.1186/s12877-019-1112-4 (PMC6444620; doi:10.1186/s12877-019-1112-4)
Supplement: Supplementary file 3 — Table S2. List of 28 quality of life items. This 28-item questionnaire was developed by the Research Group on the quality of life of older people in cantons of Vaud and Geneva (Switzerland), based on the available evidence, including the World Health Organization report on social determinants of health and the synthesis of the literature as well as the experience of the Research Group in this field. The questionnaire reflects the convergence of health, social, cultural and economic factors of older people’s QoL. (DOCX 20 kb) [file 12877_2019_1112_MOESM3_ESM.docx]

**Supplementary Table 1.** Comparison of gender and birth year distributions between the population of Lausanne and participants to the Lc65+ study in 2016

|  | **Population of Lausanne  (born in 1934-1938) ^a^** | |  | **Lc65+ study (pre-war cohort)** | |  | **P ^b^** |
| --- | --- | --- | --- | --- | --- | --- | --- |
|  | **N** | **%** |  | **N** | **%** |  |  |
| **Gender** |  |  |  |  |  |  |  |
| Women | 2151 | 62.9% |  | 601 | 61.7% |  | 0.449 |
| Men | 1270 | 37.1% |  | 373 | 38.3% |  |  |
| **Birth year (women)** |  |  |  |  |  |  |  |
| 1934 | 407 | 18.9% |  | 111 | 18.5% |  | 0.981 |
| 1935 | 447 | 20.8% |  | 128 | 21.3% |  |  |
| 1936 | 423 | 19.7% |  | 122 | 20.3% |  |  |
| 1937 | 418 | 19.4% |  | 117 | 19.5% |  |  |
| 1938 | 456 | 21.2% |  | 123 | 20.5% |  |  |
| **Birth year (men)** |  |  |  |  |  |  |  |
| 1934 | 205 | 16.1% |  | 66 | 17.7% |  | 0.733 |
| 1935 | 263 | 20.7% |  | 80 | 21.4% |  |  |
| 1936 | 270 | 21.3% |  | 77 | 20.6% |  |  |
| 1937 | 254 | 20.0% |  | 78 | 20.9% |  |  |
| 1938 | 278 | 21.9% |  | 72 | 19.3% |  |  |

^a^ Permanent resident population of Lausanne on 31^st^ December 2016 (^©^ Statistique Vaud)

^b^ Chi-squared test

**Supplementary Table 1.** (Continued)

|  | **Population of Lausanne  (born in 1939-1943) ^a^** | |  | **Lc65+ study (war cohort)** | |  | **P ^b^** |
| --- | --- | --- | --- | --- | --- | --- | --- |
|  | **N** | **%** |  | **N** | **%** |  |  |
| **Gender** |  |  |  |  |  |  |  |
| Women | 2489 | 60.3% |  | 685 | 61.3% |  | 0.476 |
| Men | 1640 | 39.7% |  | 432 | 38.7% |  |  |
| **Birth year (women)** |  |  |  |  |  |  |  |
| 1939 | 434 | 17.4% |  | 113 | 16.5% |  | 0.319 |
| 1940 | 476 | 19.1% |  | 127 | 18.5% |  |  |
| 1941 | 501 | 20.1% |  | 128 | 18.7% |  |  |
| 1942 | 512 | 20.6% |  | 138 | 20.1% |  |  |
| 1943 | 566 | 22.7% |  | 179 | 26.1% |  |  |
| **Birth year (men)** |  |  |  |  |  |  |  |
| 1939 | 267 | 16.3% |  | 74 | 17.1% |  | 0.842 |
| 1940 | 305 | 18.6% |  | 78 | 18.1% |  |  |
| 1941 | 325 | 19.8% |  | 88 | 20.4% |  |  |
| 1942 | 352 | 21.5% |  | 98 | 22.7% |  |  |
| 1943 | 391 | 23.8% |  | 94 | 21.8% |  |  |

^a^ Permanent resident population of Lausanne on 31^st^ December 2016 (^©^ Statistique Vaud)

^b^ Chi-squared test

**Supplementary Table 1.** (Continued)

|  | **Population of Lausanne  (born in 1944-1948) ^a^** | |  | **Lc65+ study (baby boom cohort)** | |  | **P ^b^** |
| --- | --- | --- | --- | --- | --- | --- | --- |
|  | **N** | **%** |  | **N** | **%** |  |  |
| **Gender** |  |  |  |  |  |  |  |
| Women | 2908 | 57.7% |  | 795 | 56.1% |  | 0.213 |
| Men | 2132 | 42.3% |  | 623 | 43.9% |  |  |
| **Birth year (women)** |  |  |  |  |  |  |  |
| 1944 | 573 | 19.7% |  | 149 | 18.7% |  | 0.618 |
| 1945 | 542 | 18.6% |  | 136 | 17.1% |  |  |
| 1946 | 558 | 19.2% |  | 152 | 19.1% |  |  |
| 1947 | 635 | 21.8% |  | 186 | 23.4% |  |  |
| 1948 | 600 | 20.6% |  | 172 | 21.6% |  |  |
| **Birth year (men)** |  |  |  |  |  |  |  |
| 1944 | 400 | 18.8% |  | 127 | 20.4% |  | 0.532 |
| 1945 | 420 | 19.7% |  | 132 | 21.2% |  |  |
| 1946 | 449 | 21.1% |  | 118 | 18.9% |  |  |
| 1947 | 426 | 20.0% |  | 124 | 19.9% |  |  |
| 1948 | 437 | 20.5% |  | 122 | 19.6% |  |  |

^a^ Permanent resident population of Lausanne on 31^st^ December 2016 (^©^ Statistique Vaud)

^b^ Chi-squared test
